# Divided method of intercostal nerve block reduces ropivacaine dose by half in thoracoscopic pulmonary resection while maintaining the postoperative pain score and 4-h mobilization: a retrospective study

**DOI:** 10.1007/s00540-023-03229-w

**Published:** 2023-08-10

**Authors:** Aiko Nakai, Jyunya Nakada, Yusuke Takahashi, Noriaki Sakakura, Katuhiro Masago, Sakura Okamoto, Hiroaki Kuroda

**Affiliations:** 1 Department of Anesthesiology, Aichi Cancer Center Hospital, 1-1 Kanokoden, Chikusa-Ku, Nagoya, 464-8681 Japan; 2 Department of Thoracic Surgery, Aichi Cancer Center Hospital, Nagoya, Japan; 3 Department of Pathology and Molecular Diagnostics, Aichi Cancer Center, Nagoya, Japan

**Keywords:** Ropivacaine, Intercostal nerve block, Early mobilization, Numerical rating scale, Thoracoscopic surgery

## Abstract

**Purpose:**

This study investigated whether the divided method of multi-level intercostal nerve block (ML-ICB) could reduce the ropivacaine dose required during thoracoscopic pulmonary resection, while maintaining the resting postoperative pain scores.

**Methods:**

This retrospective, single-cohort study enrolled 241 patients who underwent thoracoscopic pulmonary resection for malignant tumors between October 2020 and March 2022 at a cancer hospital in Japan. ML-ICB was performed by surgeons under direct vision. The differences in intraoperative anesthetic use and postoperative pain-related variables at the beginning and end of surgery between group A (single-shot ML-ICB; 0.75% ropivacaine, 20 mL at the end of the surgery) and group B (divided ML-ICB, performed at the beginning and end of surgery; 0.25% ropivacaine, 30 mL total) were assessed. The numerical rating scale (NRS) was used to evaluate pain 1 h and 24 h postoperatively.

**Results:**

Intraoperative remifentanil use was significantly lower in group B (14.4 ± 6.4 μg/kg/h) than in group A (16.7 ± 8.4 μg/kg/h) (*P* = 0.02). The proportion of patients with NRS scores of 0 to 3 at 24 h was significantly higher in group B (85.4%, 106/124) than in group A (73.5%, 86/117) (*P* = 0.02). The proportion of patients not requiring postoperative intravenous rescue drugs was significantly higher in group B (78.2%, 97/124) than in group A (61.5%, 72/117) (*P* < 0.01).

**Conclusion:**

The divided method of ML-ICB could reduce the intraoperative remifentanil dose, decrease the postoperative pain score at 24 h, and curtail postoperative intravenous rescue drug use, despite using half the total ropivacaine dose intraoperatively.

## Introduction

**Fig. 1:**
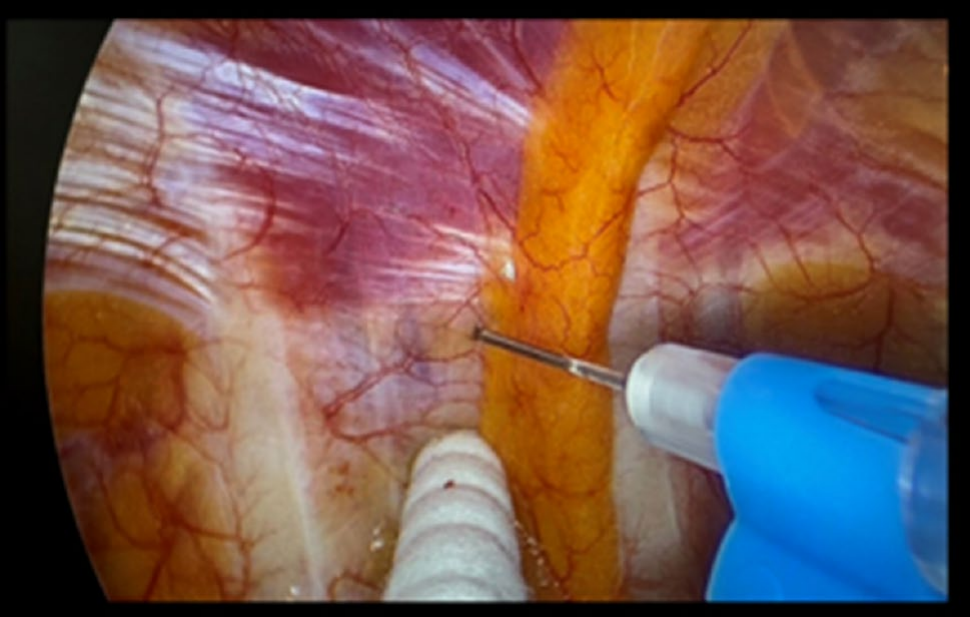
Representative schema of multi-level intercostal nerve block in thoracic surgery

The advantages of thoracoscopic surgery (TS) over open thoracotomy include faster recovery, reduced perioperative pain, and decreased postoperative morbidity [1–3]. Epidural anesthesia was previously considered the gold standard for perioperative analgesia after TS [4–6]. However, Bolotin et al. and Ahmed et al. suggested that a single-injection intercostal nerve block (ICB) is associated with reduced opioid consumption compared with systemic analgesia in adults undergoing thoracic surgery [7, 8]. In our hospital, multi-level ICB (ML-ICB) is the preferred technique for thoracic surgeons during TS, as the surgeons can visualize the intercostal and sympathetic nerves through a monitor while administering the nerve block from the thoracic cavity (Fig. 1). The paravertebral block has been shown to be superior to other locoregional analgesic techniques for the management of perioperative pain [9, 10]. Furthermore, although the timing of nerve blockade in patients undergoing gynecological, breast, gastrointestinal, and plastic surgeries has been discussed previously [11, 12], few studies have investigated the optimal intraoperative analgesia strategy, including ICB, for TS. Therefore, in this study, the divided method of ML-ICB (at the beginning and the end of surgery) was selected to compensate for the inferior effect of ICB in the management of perioperative pain since locoregional analgesia is delivered before the patient experiences pain (preemptive anesthesia).

The null hypothesis was that the divided ML-ICB method with half-dose ropivacaine is equivalent to single-shot ML-ICB with respect to the dosage of perioperative analgesics. This effect would benefit our TS protocol, which has enabled early postoperative mobilization and chest drain removal within 4 h after surgery (4-h mobilization) since January 2017 [13].

## Methods

This retrospective, single-center study reviewed 281 patients who underwent TS for malignant tumor resection at our hospital between October 2020 and March 2022. Patients aged 18 years and older with an American Society of Anesthesiologists (ASA) grade ranging from 1 to 3 were included. The exclusion criteria were as follows: (a) patients with benign lesions; (b) patients who underwent emergency surgery; (c) patients with a history of anaphylaxis or allergy to local anesthetics; (d) presence of an implantable cardioverter defibrillator (ICD) or pacemaker; (e) patients with incomplete data; and (f) patients who did not receive ML-ICB. This study adhered to the principles of the Declaration of Helsinki. The study protocol was approved by our hospital’s institutional review board (IRB) (approval number: 2021–0-135; date of IRB approval: December 29, 2021; study duration: April 1, 2019, to March 31, 2022). The need for informed consent was waived owing to the study’s retrospective nature.

### Surgical and anesthetic technique

The TS procedure entailed a single or multiple (3 or 4)-port approach, and the port level was determined according to the pneumonectomy site. The length of one incision was extended to 3–5 cm to extract the resected lung. During the nerve block the thoracoscopic screen was shared by the surgeons, anesthesiologists, and nurses. The surgeon performed ML-ICB and local anesthesia according to the following protocol.

*Group A: Single-shot method* (October 2020 to March 2021) Before closure of the wound, ML-ICB was performed under direct visualization of the thoracic cavity using a 22-G Catherine needle (TERUMO, Japan; 0.75% ropivacaine 20 mL). The injection site was the inferior edge of the ribs, 6 to 8 cm lateral to the erector spinae. The needle tip was located just below the visceral pleura avoiding the surrounding blood vessels (Fig. 1). Five or six intercostal nerve levels were blocked at port level ± 1. The total dose of ropivacaine was 150 mg.

*Group B: Divided method* (April 2021 to March 2022).

Preoperative local anesthetic was injected around the incision site with a 22-G standard needle (0.25% ropivacaine 4 mL). ML-ICB was performed after inserting the ports into the thoracic cavity with a 22-G winged needle (0.25% ropivacaine 6 mL) using the same method as group A. Before closure of the wound, additional ML-ICB was performed (0.25% ropivacaine 20 mL). The total dose of ropivacaine was 75 mg, i.e., half that of the dose administered to group A.

All patients were managed under general anesthesia with continuous monitoring. The choice of inhalation or intravenous anesthetic, including steroids, ketamine hydrochloride, midazolam, droperidol, acetaminophen, and nonsteroidal anti-inflammatory drugs was not standardized and was left to the anesthesiologists’ discretion. Rescue analgesics (acetaminophen, nonsteroidal anti-inflammatory drugs, or tramadol) were used in the ward to facilitate early ambulation (4-h mobilization) according to the Numerical Rating Scale (NRS) score and patient demands. Postoperative complications, such as nausea, vomiting, prolonged paresthesia, and pain, were reported by the nursing staff. There were no differences in the perioperative analgesic treatment, surgical procedure, or post-surgical protocol between the two groups.

This study evaluated postoperative NRS scores at 1 h and 24 h, in addition to the dose of perioperative opioids, including intravenous rescue analgesics, and the proportion of patients who achieved 4-h mobilization. We designated an NRS score of 3 as the cut-off value, where a score equal to or less than 3 corresponded to “none to light pain” and scores from 4 to 10 corresponded to “mild to severe pain.” We also collected information on the following parameters: clinical characteristics (age, sex, and body mass index) and physical status according to the ASA classification.

### Statistical analysis

Statistical analyses were performed using JMP 16.2.0 (2020–2021 SAS Institute Inc, Cary, NC). Continuous variables were presented as the mean and standard deviation, and categorical variables were presented as the frequency and percentage. The chi-square test and multivariable logistic regression analysis were used to calculate the correlations between categorical variables. The two-tailed Student’s *t*–exact test was used to compare any two variables. Statistical significance was set at *P* < 0.05.

## Results

### Patient characteristics

**Fig. 2:**
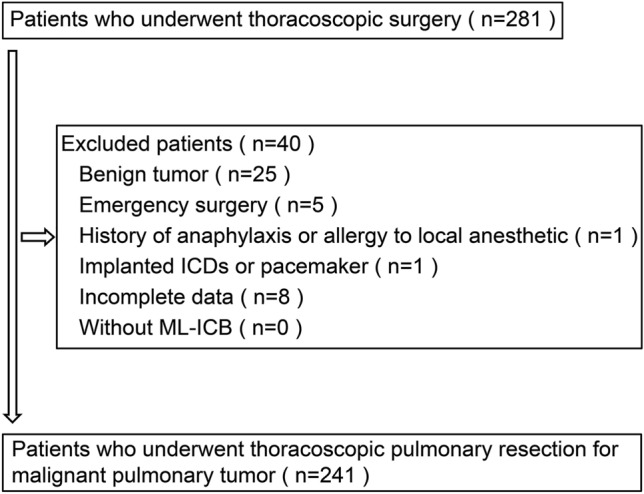
Patient flow chart illustrating the enrollment process in this study

Forty patients were excluded for the following reasons (Fig. 2): 25 had benign tumors, five underwent emergency surgery, one had a history of local anesthetic allergy, one had an implanted ICD or pacemaker, and eight had incomplete data; all patients were managed with ML-ICB. Thus, the study included 241 patients: group A comprised 117 patients (48.5%) and group B comprised 124 patients (51.45%). The clinical characteristics and surgical procedure of patients who underwent TS pulmonary resection did not differ significantly between the groups (Table 1). Additionally, no patient had severe comorbidities, such as chronic heart failure, chronic kidney disease requiring dialysis, or liver cirrhosis of Child–Pugh Class B or higher.

**Table 1:** Patient characteristics

Variables	Group A N = 117	Group B N = 124
Age (years, mean)	66	66
Male (n, %)	63 (53.8%)	67 (54.0%)
BMI	22.4 ± 3.17	22.4 ± 3.74
ASA PS Classification		
Class I (n, %)	19 (16.2%)	16 (12.9%)
Class II (n, %)	90 (76.9%)	101 (81.4%)
Class III (n, %)	8 (6.8%)	7 (5.6%)
Surgery Type		
Pneumonectomy (n, %)	0 (0%)	0 (0%)
Lobectomy (n, %)	64 (54.7%)	55 (44.3%)
Segmentectomy (n, %)	41 (35%)	50 (40.3%)
Partial resection (n, %)	12 (10.2%)	19 (15.3%)

Values are presented as mean (standard deviation) or number (proportion)

*ASA PS* American Society of Anesthesiologists physical status, *BMI* body mass index

### Intra- and postoperative outcomes

Table 2 summarizes the intra- and postoperative outcomes. The intraoperative use of remifentanil was significantly lower in group B (14.4 ± 6.4 μg/kg/h) than in group A (16.6 ± 8.4 μg/kg/h) (*P* = 0.02). However, the dose of fentanyl administered to patients did not differ significantly between the groups (group A vs. group B: median, 2.3 μg/kg/h and 2.0 μg/kg/h, respectively) (*P* = 0.12). Additionally, there was no significant difference in the intraoperative use of inhalation or intravenous anesthesia and acetaminophen, surgical time, and bleeding between the groups (group A vs. group B: 27 (23%) vs. 24 (19.3%), 111 (94.8%) vs. 115 (92.7%), 89 (76.0%) vs. 93 (75.6%), 158.8 ± 53.6 min vs. 151.0 ± 55.9 min, 8.3 ± 11.0 mL vs. 7.9 ± 9.2 mL). Any unexpected adverse events of vascular puncture, as well as the incidence of local anesthetic systemic toxicity, was recorded. Major vascular injuries, local anaesthetic systemic toxicity, and prolonged paraesthesia were not reported during ML-ICB (*P* > 0.99).

**Table 2:** Intra- and postoperative outcomes

	Group A N = 117	Group B N = 124	*P*-value
Intraoperative use of remifentanil (μg/kg/h)	16.7 ± 8.4	14.4 ± 6.4	0.02*
Intraoperative use of fentanyl (μg/kg/h)	2.3 ± 1.3	2.0 ± 1.3	0.12
Vascular puncture (number, %)	0 (0)	0 (0)	> 0.99
Paresthesia (number, %)	0 (0)	0 (0)	> 0.99
LAT (number, %)	0 (0)	0 (0)	> 0.99
Surgical time (min)	158.8 ± 53.6	151.0 ± 55.9	0.26
Bleeding (mL)	8.3 ± 11.0	7.9 ± 9.2	0.47
Inhalation anesthesia (n, %)	27 (23.0%)	24 (19.3%)	0.47
Total intravenous anesthesia (n, %)	111 (94.8%)	115 (92.7%)	0.49
Intraoperative use of acetaminophen (n, %)	89 (76.0%)	93 (75.6%)	0.93

The data are presented as the mean ± SD

^*^Indicates statistical significance, *P* < 0.05

*SD* standard deviation, *LAT* local anesthetic toxicity

Table 3 shows that the proportion of patients with an NRS score of 0 to 3 at 1 h postoperatively was higher in group B (102/124, 82.2%) than in group A (87/117, 74.3%); however, the difference was not significant (*P* = 0.13). The proportion of patients with an NRS score of 0 to 3 at 24 h was significantly higher in group B (85.4%, 106/117) than in group A (73.5%, 86/117) (*P* = 0.02). The proportion of patients who did not require postoperative intravenous rescue drug (acetaminophen, nonsteroidal anti-inflammatory drugs, or tramadol) was also significantly higher in group B (78.2%, 97/124) than in group A (61.5%, 72/117) (*P* < 0.01). The proportion of patients who achieved 4-h mobilization was 90.3% (112/117) in group A and 87.1% (108/124) in group B (*P* = 0.43).

**Table 3:** Patient outcomes: pain-related parameters and ambulation

	Group A N = 117	Group B N = 124	*P*-value
NRS score of 0–3 at 1 h	87 (74.3%)	102 (82.2%)	0.13
NRS score of 0–3 at 24 h	86 (73.5%)	106 (85.4%)	0.02*
No intravenous rescue drug use	72 (61.5%)	97 (78.2%)	< 0.01*
Successful 4-h mobilization	112 (90.3%)	108 (87.1%)	0.43

The data are presented as number (%)

^*^Indicates statistical significance, *P* < 0.05

*NRS* numerical rating scale

## Discussion

The principal findings of this study can be summarized as follows: (a) postoperative pain control at 24 h was significantly better in group B than in group A; (b) a significant reduction was noted in postoperative intravenous rescue drug usage in group B, including acetaminophen, nonsteroidal anti-inflammatory drugs, and tramadol, but this effect was not seen in group A; and (c) the intraoperative dose of remifentanil was significantly lower in group B than in group A.

Until March 2021, there had been concerns that full dose ropivacaine (150 mg: 0.75% ropivacaine 20 mL) at the end of surgery may be excessive. Therefore, from April 2021, anesthesiologists suggested the divided ML-ICB protocol, where one-sixth of the total dose (25 mg: 10 mL 0.25% ropivacaine) is administered at the beginning of TS as preemptive analgesia, and the remaining one-third dose (50 mg: 20 mL 0.25% ropivacaine) is administered after deducting half the previous dose at termination (total dose 75 mg: 30 mL 0.25% ropivacaine).

Administering preemptive locoregional analgesia attenuates postoperative pain [14–16], decreases supplemental analgesic requirement, and prolongs the average time to the first use of rescue analgesic drugs in various procedures. Moreover, Lee et al. showed that the pre-incisional thoracic paravertebral block conferred a significant preemptive visceral analgesic effect and significantly reduced the amount of postoperative opioid consumption [16]. Therefore, the divided method was chosen in our facility. In this study, the intraoperative use of remifentanil was significantly lower in group B than in group A, and the proportion of patients with an NRS score of 0 to 3 24 h postoperatively was significantly higher in group B (85.4%, 106/117) than in group A (73.5%, 86/117). These results imply that preemptive locoregional analgesia is essential for the amelioration of perioperative pain. However, there was no significant difference in postoperative pain at 1 h or in the number of patients receiving intravenous acetaminophen intraoperatively between the two groups (group A vs. group B: 76% vs. 75.6%), which may potentially contribute to early postoperative pain. Moreover, the proportion of patients who successfully achieved 4-h mobilization was 90.3% (112/117) in group A and 87.1% (108/124) in group B, despite reducing the ropivacaine dose by half in group B.

The widespread use of opioids to relieve acute postoperative pain has masked its perverse effects on the surgical procedure itself [17–19]. The well-known adverse effects of opioids may result in delayed recovery and adversely affect patients by causing deep sedation and respiratory depression [18]. Some studies have highlighted the dark side of opioids: neuroadaptation prevents the ability of opioids to provide long-term analgesia and produces the opposite effect (opioid-induced hyperalgesia) [17]. No significant difference in the intraoperative use of fentanyl was observed between the two groups in the present study. Nevertheless, the intraoperative use of remifentanil and postoperative intravenous use of rescue drugs (including opioids) were higher in group A than in group B. Thus, preemptive locoregional analgesia could possibly help reduce the adverse effects of opioids. Optimized ML-ICB may improve the patients’ quality of life and potentially reduce opioid dependency and healthcare costs.

This study has several limitations. First, this was a retrospective, single-center study, and unknown confounding factors may have influenced the results. Second, pain is often devastating for the affected individuals, and it is not easy to numerically express the magnitude of pain. Third, there was no unified anesthetic protocol, and total intravenous anesthesia may have impacted patient recovery and the perioperative fentanyl dose.

In conclusion, the divided method of ML-ICB (pre-emptive analgesia) could reduce the intraoperative use of remifentanil, decrease the 24-h NRS score, and curtail the use of postoperative intravenous rescue drugs, despite reducing the ropivacaine dose by half. Prospective studies that eliminate confounders and further investigate the optimal ropivacaine dose are required to validate these results.

## Data Availability

The data that support the findings of this study are available on request from the corresponding author. The data are not publicly available due to restrictions (i.e., they contain information that could compromise the privacy of research participants).
