# Correction to: Divided method of intercostal nerve block reduces ropivacaine dose by half in thoracoscopic pulmonary resection while maintaining the postoperative pain score and 4-h mobilization: a retrospective study

**DOI:** 10.1007/s00540-023-03266-5

**Published:** 2023-10-04

**Authors:** Aiko Nakai, Jyunya Nakada, Yusuke Takahashi, Noriaki Sakakura, Katuhiro Masago, Sakura Okamoto, Hiroaki Kuroda

**Affiliations:** 1 Department of Anesthesiology, Aichi Cancer Center Hospital, 1-1 Kanokoden, Chikusa-Ku, Nagoya, 464-8681 Japan; 2 Department of Thoracic Surgery, Aichi Cancer Center Hospital, Nagoya, Japan; 3 Department of Pathology and Molecular Diagnostics, Aichi Cancer Center, Nagoya, Japan

**Correction to: Journal of Anesthesia (2023) 37:749–754** 10.1007/s00540-023-03229-w

In the original publication of the article, under the section “Statistical analysis”, the following sentence was published incorrectly as “The chi-square test and multivariate logistic regression analysis were used to calculate the correlations between categorical variables”. The correct sentence should read as follows, “The chi-square test was used to calculate the correlations between categorical variables”.

